# Flexible Artificial Optoelectronic Synapse based on Lead‐Free Metal Halide Nanocrystals for Neuromorphic Computing and Color Recognition

**DOI:** 10.1002/advs.202202123

**Published:** 2022-06-05

**Authors:** Ying Li, Jiahui Wang, Qing Yang, Guozhen Shen

**Affiliations:** ^1^ State Key Laboratory for Superlattices and Microstructures Institute of Semiconductors, Chinese Academy of Sciences Beijing 100083 China; ^2^ Department of Chemistry and Laboratory of Nanomaterials for Energy Conversion University of Science and Technology of China Hefei 230026 P. R. China; ^3^ School of Integrated Circuits and Electronics Beijing Institute of Technology Beijing 100081 China

**Keywords:** color recognition, Cs_3_Bi_2_I_9_, flexible artificial synapse, lead‐free halide perovskite, neuromorphic computing

## Abstract

Optoelectronic synapses combining optical‐sensing and synaptic functions are playing an increasingly vital role in the neuromorphic computing systems development, which can efficiently process visual information and complex recognition, memory, and learning. Metal halides are considered promising candidates for synaptic devices due to their excellent optoelectronic properties. However, the toxicity of lead and the further development of device functions are the recognized problems at present. Herein, a flexible optoelectronic synapses system based on high‐quality lead‐free Cs_3_Bi_2_I_9_ nanocrystals is demonstrated, in which the carrier confinement caused by the band mismatching between the Cs_3_Bi_2_I_9_ and the organic semiconductor layer provides the possibility to simulate synaptic behaviors. The synaptic functions including long/short‐term memory and learning‐forgetting‐relearning are demonstrated in this device and visual perception, visual memory, and color recognition functions are successfully implemented. Additionally, the flexible device exhibits excellent robustness and can realize imaging of light distribution under curved hemispheres similar to the human eye. Finally, through the simulation based on an artificial neural network algorithm, the device successfully realizes the high‐precision recognition of handwritten digital images and possesses a strong fault tolerant capability even in bending states. These results are expected to drive the practical progress of metal halide for neuromorphic computing.

## Graphical abstract

A flexible optoelectric artificial synapse based on Cs_3_Bi_2_I_9_ nanocrystals is demonstrated. The synaptic functions including long/short‐term memory and learning‐forgetting‐relearning are demonstrated. And the applications related to visual perception‐memory and color recognition are discussed. Finally, through the simulation based on an artificial neural network algorithm, the image recognition is successfully realized and possesses a strong fault tolerance even in bending states.

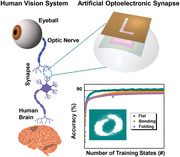

## 1 Introduction

In the era of artificial intelligence, traditional computing system based on the Von Neumann architecture is facing significant challenges such as the inherent upper limit of computing speed and the sharp increase in energy consumption, which can be attributed to the separation of processing and memory units.^[^ ^1^, ^2^, ^3^, ^4^ ^]^ In order to overcome the predicament, researchers have been trying to find a more efficient system. Among these, neuromorphic computing based on artificial synaptic shows great advantages of high‐speed computing, and ultra‐low power consumption for perception, learning, and memory, and is expected to become an important method to solve the above problems.^[^ ^5^, ^6^, ^7^, ^8^, ^9^ ^]^ Compared with neuromorphic devices regulated by electrical signals that have been widely studied, the optoelectronic synaptic devices contain many remarkable characteristics such as low crosstalk, high interference immunity, and low power consumption, which is more appropriate for ultrahigh‐speed computing.^[^ ^10^, ^11^, ^12^ ^]^ Moreover, the development of artificial vision with flexibility, complexity, and adaptability brought about by the research of light‐stimulated synaptic device will play a vital role in human life. Recently, organic materials,^[^ ^12^, ^13^, ^14^ ^]^ metal oxides,^[^ ^15^, ^16^, ^17^, ^18^ ^]^ graphene,^[^ ^19^, ^20^ ^]^ MoS_2_,^[^ ^6^, ^21^ ^]^ WSe_2_,^[^ ^22^ ^]^ and other 2D materials have been employed to fabricate optoelectronic synapses, showing remarkable performances. However, many of these materials require complex and expensive preparation methods, which are not beneficial to the progress of large‐scale and low‐cost flexible synaptic devices.

As one of the candidate materials for photoactive materials in optoelectronic synapses, metal halides have attracted tremendous attention due to their remarkable characteristics such as high and well‐balanced carrier transport ability, high light absorption coefficient, and low‐temperature processing technique.^[^ ^23^, ^24^ ^]^ Existing work principles include the use of floating gate transistor,^[^ ^25^, ^26^, ^27^, ^28^ ^]^ the adjustment of the ratio of vacancies in the metal halide materials,^[^ ^29^, ^30^ ^]^ as well as the carriers trapping and de‐trapping at the heterojunction interface that has been actively investigated to realize ideal synaptic device.^[^ ^31^, ^32^, ^33^ ^]^ Among these, the proposed floating gate transistor device has attracted a lot of attention because of its advantages of clear operation mechanism, controllable testing parameters, and concurrent learning.^[^ ^25^, ^26^, ^27^, ^28^, ^29^, ^30^, ^31^, ^32^, ^33^, ^34^ ^]^ Up to now, the reported floating gate optoelectronic synapses based on metal halide are mainly focused on lead halide perovskites, and fundamental synaptic functions have been simulated, such as paired‐pulse facilitation (PPF), short/long‐term plasticity and the transition from short‐term plasticity to long‐term plasticity.^[^ ^25^, ^26^ ^]^ Despite the progress that has been made, the toxicity and environmental instability have cast a gloomy shadow over their further applications. And most of reported works focus on the implementation of basic synaptic properties, and rarely involve further development of device functions. In addition, with the rapid development of wearable electronics, there is an increasing demand for flexible devices with ultralow power consumption, robust bending stability, and strong data processing capabilities. Therefore, searching low‐toxic flexible synaptic device and expanding the research applications to address the above hurdles is imperative, but no such report can be found yet.

In this work, we demonstrate a flexible optoelectronic synaptic transistor based on high‐quality lead‐free Cs_3_Bi_2_I_9_ nanocrystals (NCs) and organic semiconductor, comprising Al_2_O_3_/Cs_3_Bi_2_I_9_ NCs‐PMMA (polymethyl methacrylate)/DPPDTT (poly[2,5‐(2‐octyldodecyl)‐3,6‐diketopyrrolopyrrole‐alt‐5,5‐(2,5‐di(thien‐2‐yl)thieno[3,2‐*b*]thiophene)]) layer and Au source–drain electrodes. Synaptic functions such as excitatory postsynaptic current (EPSC), PPF, synaptic plasticity, and “learning‐forgetting‐learning” were successfully performed. In addition, the prepared array shows remarkable flexibility and robustness with no significant change in current after bending for 1000 times. On this basis, the prepared flexible synaptic arrays can effectively conduct learning and memory simultaneously, and successfully recognize images of different colors of light (405, 532, and 635 nm) under low light intensity. Finally, the device successfully realizes the recognition application of handwritten digital images through the simulation based on an artificial neural network (ANN) algorithm. The results show that the digital images recognition has high accuracy and strong fault tolerance under bending states, which further prove the potential in the field of neuromorphic computing. This work provides an efficient way for applying artificial synaptic devices to neuromorphic networks.

## 2 Results and Discussion

**Figure 1:**
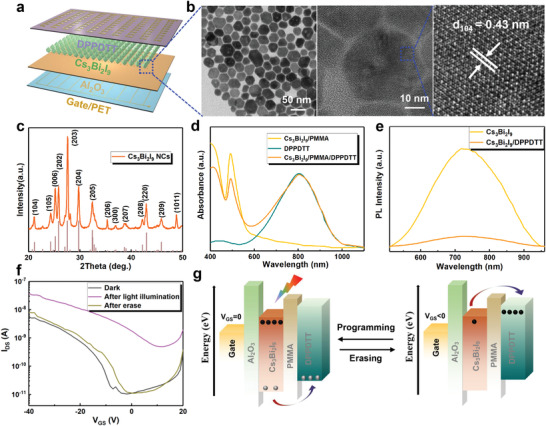
a) Schematic diagram of the Cs_3_Bi_2_I_9_ NCs‐based optoelectronic synapses array device. b) TEM and HRTEM image and c) XRD patterns of the Cs_3_Bi_2_I_9_ NCs. d) Absorption spectra of the Cs_3_Bi_2_I_9_ NCs/PMMA, DPPDTT, and Cs_3_Bi_2_I_9_ NCs/PMMA/DPPDTT films. e) PL spectra of the Cs_3_Bi_2_I_9_ NCs/PMMA and Cs_3_Bi_2_I_9_ NCs/PMMA/DPPDTT films. f) Transfer curves of the device. g) Schematic energy diagram of the device.

An artificial synaptic array device based on Cs_3_Bi_2_I_9_ NCs field effect transistor (FET) was designed and studied in detail, and the structure is shown in **Figure** **1**a. The Cs_3_Bi_2_I_9_ NCs/PMMA composite films were prepared on polyethylene terephthalate (PET) substrates with specific shape electrodes by spin‐coating and used as hybrid floating gate/tunneling dielectric layers. Organic material DPPDTT possesses high air stability and high charge mobility,^[^ ^35^ ^]^ and was prepared on the top of the Cs_3_Bi_2_I_9_ NCs/PMMA composite film as a semiconductor channel. Cr/Au electrodes which nested with the bottom gate were deposited on the DPPDTT as source and drain. Specially, hexagonal Cs_3_Bi_2_I_9_ NCs were prepared via a modulated colloidal synthetic route, which simplified the synthesis steps compared to the existing hot‐injection method and solved the problem of suboptimal size control.^[^ ^36^, ^37^ ^]^ In brief, trimethylsilyl iodide (TMSI) was injected in the dissolved solution of cesium acetate and bismuth acetate. After the injection of the TMSI at 100 ℃ for 10 s, the color changes from yellow brown to bright orange‐red color indicating the nucleation of the Cs_3_Bi_2_I_9_ NCs. Transmission electron microscope (TEM) images of synthesized Cs_3_Bi_2_I_9_ NCs are shown in Figure 1b, which are characterized as uniformly distributed hexagonal Cs_3_Bi_2_I_9_ NCs. The diameter is in the range of 15.52–41.66 nm with an average size of 26.94 ± 5.56 nm (Figure S1, Supporting Information). A well‐defined crystalline structure can be observed from the high‐resolution TEM (HRTEM). The characteristic distance of lattice plane is 0.43 nm, which corresponds to the (104) crystal plane of hexagonal Cs_3_Bi_2_I_9_. Figure 1c shows X‐ray diffraction (XRD) patterns of the synthesized Cs_3_Bi_2_I_9_ NCs. A set of diffraction peaks at 21.027°, 27.567°, 29.747°, 32.347°, 35.307°, and 38.607° can be assigned to the (104), (203), (204), (205), (206), and (207) planes of the hexagonal Cs_3_Bi_2_I_9_ (space group *P6_3_/mmc*, *a* = 8.4116 Å, *b* = 8.4116 Å, *c* = 21.1820 Å), which further proved the pure hexagonal‐phase structure of Cs_3_Bi_2_I_9_ NCs. In order to verify the stability of Cs_3_Bi_2_I_9_ NCs, XRD and TEM measurements were further performed after storage for 30 days in air ambient (20 ℃, 35−50% humidity). It can be observed from Figure S2 in the Supporting Information that the samples maintain the structure integrity without decomposition after 30 days storage. And no other diffraction signals were found. In addition, the TEM morphology did not change significantly before and after storage for 30 days (Figure S3, Supporting Information). The results demonstrate good stability of the Cs_3_Bi_2_I_9_ NCs against environment oxygen/moisture, which proves that the photoactive material has good practicability.

Then, preliminary optical properties of Cs_3_Bi_2_I_9_/PMMA, DPPDTT, Cs_3_Bi_2_I_9_/PMMA/DPPDTT are investigated (Figure 1d,e). The UV‐visible absorption spectra of Cs_3_Bi_2_I_9_/PMMA possesses a strong absorption below 600 nm, and the corresponding Tauc plot of the absorption spectra (Figure S4, Supporting Information) reveals that Cs_3_Bi_2_I_9_ NCs possesses an indirect bandgap of 2.20 eV, which is consistent with previous reports.^[^ ^38^, ^39^ ^]^ The DPPDTT film shows a strong absorption above 600 nm. In addition, through the comparison of steady‐state photoluminescence (PL) spectra, the charge‐transfer efficiency was probed. As shown in Figure 1e, the Cs_3_Bi_2_I_9_/PMMA films reveal a broad and strong PL emission peak at 730 nm. On the contrary, the hybrid films exhibit a weak spectrum, and the integral intensity decreased by ≈84%, indicating that the photogenerated carriers are transferred in the hybrid structure. Figure 1f shows the transfer characteristics of the device, which is performed to assess the charge‐storage effectiveness before and after light irradiation (405 nm, 2.50 mW cm^−2^, duration of 20 s) at a *V*_DS_ of −10 V. The threshold voltage (*V*_th_) shifts in the positive direction after light illumination operation, indicating that the device can not only act as the electron capture sites but also store the trapped charges even after the irradiation is removed.^[^ ^25^, ^40^ ^]^ When the electrical pulse erase of negative *V*_GS_ is applied (−10 V, 1 s), the *V*_th_ can be restored to its initial state, proving that the trapped electrons are released. The output curves of the FET under dark condition were studied, as shown in Figure S5 in the Supporting Information. It can be observed from the curve that with the increase of the negative *V*_GS_, the *I*_DS_ gradually increases, which proves that the *V*_GS_ can be used to regulate the charge carrier transport. In addition, we investigated the output curve under different wavelengths of light (Figure S6, Supporting Information). Compared with *I*_DS_ under dark condition, the current increases with light illumination, indicating that light can regulate the carriers transport effectively. And the change of *I*_DS_ at different wavelengths (405, 532, 635 nm) is consistent with the change of absorption spectra.

Based on the above results, we believe that the operating mechanism of the device for light‐writing and electrical‐erasing can be explained by the energy band diagrams. Based on the UV photoelectron spectroscopy (Figure S7, Supporting Information) and UV‐visible absorption spectra, the energy band alignment of the Cs_3_Bi_2_I_9_/DPPDTT is plotted in Figure 1g. Here, the conduction band minimum and valence band maximum of Cs_3_Bi_2_I_9_ layer are −4.19 and −6.39 eV, and those of DPPDTT are −3.50 and −5.20 eV, respectively.^[^ ^33^, ^41^ ^]^ Under illumination, a large number of photocarriers are generated in Cs_3_Bi_2_I_9_ NCs. The photogenerated holes are transferred to the DPPDTT by the bending of energy band, and the photogenerated electrons are trapped in the conduction band due to band mismatching between the Cs_3_Bi_2_I_9_ and the organic semiconductor layer. The trapped photogenerated electrons can be used as an additional electric filed to accelerate the holes escape into the DPPDTT layer. In addition, since the Cs_3_Bi_2_I_9_ NCs in the Cs_3_Bi_2_I_9_ NCs/PMMA films is in a discrete state, the trapped electrons are further retained, and the dielectric layer also effectively suppresses current dissipation.^[^ ^28^ ^]^ When the negative *V*_GS_ is applied, the trapped electrons could be electrically erased, breaking through the interfacial barrier to motivate the hole injection from DPPDTT to Cs_3_Bi_2_I_9_ NCs.

**Figure 2:**
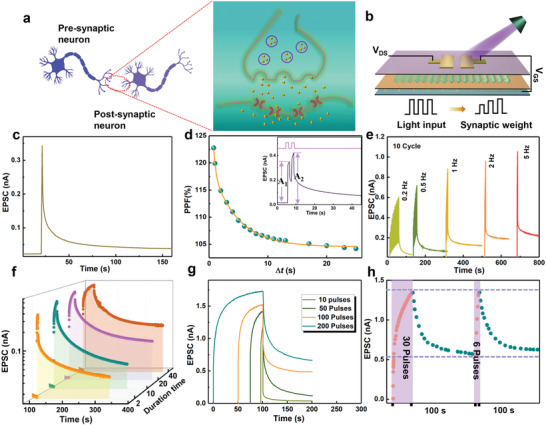
a) Schematic diagram of neurons and synapses in the brain. b) Schematic of the neuromorphic device. c) EPSC behavior triggered by a light pulse (405 nm, 0.1 mW cm^−2^, duration of 1 s). d) PPF index as a function of the time interval of light pulse pairs. The inset shows the EPSCs excited by two successive light pulses with 1 s interval (405 nm, 0.1 mW cm^−2^, duration of 1 s). e) EPSC excited by different light pulse frequency. f) EPSC curves with different light duration (2, 10, 20, and 40 s) and g) different pulses (10, 50, 100, and 200 pulses). h) Learning experience of the device (0.1 mW cm^−2^, 2 Hz).

Synapse refers to the structure in which the stimulus of one neuron is transmitted to another neuron or to another cell, as shown in **Figure** **2**a. The presynaptic neuron responds to the stimulus and then release the neurotransmitters (normally electrical and chemical synapses) to connect with the receptors in the postsynaptic neuron.^[^ ^42^ ^]^ Similar to the synapses in the human visual system, most of the synaptic devices that simulate synaptic behavior use electrical or photonic signals to supply action potentials for synapses. Figure 2b presents the elementary diagram of a single synaptic device based on Cs_3_Bi_2_I_9_ NCs/PMMA/DPPDTT FET, providing photonic signals as action potential for emulating several important synaptic functions. First, we studied the EPSC behavior under light illumination. As shown in Figure 2c, during the 1 s lighting process, the current continued to increase to 0.34 nA, and the current exhibits fast decay followed by slow decay over time when the light is turned off. After 160 s, the current increases by 173% compared with the initial value. This phenomenon is consistent with the synaptic plasticity in the human system.^[^ ^43^ ^]^ PPF effect is a form of the short‐term memory (STM), which refers to the increase rate in the amplitude of the postsynaptic current between two consecutive spikes. The inset in Figure 2d presents that the continuous EPSC behaviors are stimulated by a pair of light with the same pulse width and 1 s interval (405 nm, 0.1 mW cm^−2^). It can be seen that the second magnitude EPSC (*A*_2_) is obviously higher than the first one (*A*_1_). When the first light pulse is applied, photogenerated carriers are generated in the Cs_3_Bi_2_I_9_ NCs/PMMA films, resulting in an increase in EPSC. Due to the energy band difference of Cs_3_Bi_2_I_9_ NCs and the dielectric layer, the trapped electrons in the Cs_3_Bi_2_I_9_ NCs need to take a long time to decay after turned off the light. When the second pulse is applied, more trapped electrons induce a higher current level, so more photogenerated carriers accumulate to trigger the PPF effect. Figure 2d shows the PPF effect simulated by two identical light pulses at different time intervals, which can be described by the following relationship: PPF = *A*_2_/*A*_1_ × 100%. And the fitting result agrees well with the data (orange line). Figure 2e exhibits the EPSC change behavior of the device under different light pulse frequencies, and it can be seen that the EPSC gradually increases with the frequency, and the phenomena correspond to the biological synapse behaviors. For practical application in the optoelectronic field, study on the stability of such synaptic device is necessary. In this work, we studied the EPSC of the unencapsulated device under the same testing condition in the open air. As shown in Figure S8 in the Supporting Information, the device maintains almost the same EPSC properties after 1 month storage. In addition, the unencapsulated device retained 92% of the original current value after storage in air for 60 days (Figure S9, Supporting Information), indicating that the FET has remarkable environmental stability.

Another major feature of synaptic plasticity is STM and long‐term memory (LTM). Normally, the transition from STM to LTM can be realized through repeated and long‐time stimulation, which is known as the basis of biological memory and learning. Here, the EPSC curve changes under light pulses with different intensities are shown in Figure S10 in the Supporting Information. After 45 s decay, increases in current values of 40, 60, 90, 140 pA are observed compared with the original value (20 pA) when the lighting power density increases from 0.08, 0.10, 0.50, 2.13 mW cm^−2^. Therefore, intense light pulses stimulate the conversion of the synaptic plasticity from STM to LTM during the cumulative modulation.^[^ ^44^ ^]^ Moreover, Figure 2f,g demonstrates the transition process from STM to LTM under different light width and pulse numbers, respectively. Similarly, with the increase of the light pulse width (2 to 40 s) and pulse numbers (10 to 200), the EPSC value gradually increased after the light was removed. Based on the characteristic of the device, the “learning‐forgetting‐relearning‐forgetting” process is investigated in Figure 2h. The light‐on state represents the process of learning or relearning, and the light‐off state corresponds to the forgetting process. In the first learning process, after applied 30 light pulses to stimulate the device, the EPSC was significantly enhanced after learning. When the light is removed, the EPSC attenuates 70% of the maximum value within 100 s. This process is considered to be a process of forgetting. In the second learning process, the EPSC returns to the initial level, only 6 light pulses were applied. Similarly, the EPSC maintained a higher value than the first cycle after 100 s of decay. This phenomenon is similar to the Ebbinghaus forgetting curve. It takes less time to relearn the amnesic information, and the memory can be enhanced after relearning. Except for natural forgetting, the device can reduce the erasing time by applying negative *V*_GS_. As shown in Figure S11 in the Supporting Information, the application of *V*_GS_ accelerates the decrease of EPSC. In addition, the change of the light‐writing and electrical‐erasing current value in each cycle is small, indicating the good stability of the device.

**Figure 3:**
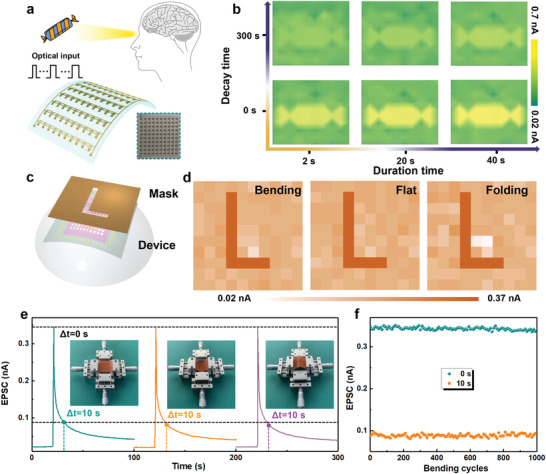
a) Schematic diagram of a human vision system and the flexible device. b) The EPSC image mapping of the 10 × 10 array under 2, 20, and 40 s light duration. c) Schematic illustration for measuring the patterned light. d) The resulting image mapping in bending, flat, and folding state. e) EPSC behavior triggered by a light pulse (405 nm, 0.1 mW cm^−2^, duration of 1 s) in flat, bending, and folding states. f) Changes in EPSC values at 0 and 10 s after light removal. The bending cycles are 1000 times and the interval is 10 times.

The proposed device exhibits excellent sensitivity to duration time, which makes it suitable for mimicking human visual perception and memory. Here, the visual perception function is emulated by a flexible 10 × 10 array. **Figure** **3**a shows the schematic structure and photograph of the flexible array. Performance uniformity and electrical stability in the bending state are crucial for imaging applications of flexible optoelectronic synapse arrays.^[^ ^45^, ^46^ ^]^ We first verified the uniformity of 100 pixels by making statistics on the current. As summarized in Figure S12 in the Supporting Information, all pixels have similar dark currents (23.86 ± 5.70 pA) and photocurrent (0.64 ± 0.02 nA) value after light illumination of 40 s (*V*_GS_ = 0 V, *V*_DS_ = −10 V, 0.1 mW cm^−2^). The results prove that all pixels of arrays possess satisfactory uniformity. Moreover, statistical dark currents were performed on 100 pixels after electrical erasing. As can be seen in Figure S13a in the Supporting Information, the device possesses outstanding current uniformity. And the corresponding statistical dark current is shown in Figure S13b in the Supporting Information, all pixels have similar dark current ranging from 10 to 40 pA, with an average value of 24.22 ± 5.69 pA. It should be noted that the average value is almost equal to the dark current before electrical erasing, implying the outstanding electrical stability of the array device. Then, to investigate the imaging ability and flexible stability, the flexible array was fixed on hemispherical support and patterned illumination was applied to the device by using a shadow mask, as shown in Figure 3c. The pixels under illumination (405 nm, 0.1 mW cm^−2^, duration of 1 s) exhibited a higher current, while the other pixels have a relatively low current level. As a result, a clear letter of L can be identified (Figure 3d). And the imaging result is consistent with those in the flat and folding states, which indicates remarkable flexible stability under the curved hemispheres. Furthermore, the synaptic behaviors with bending and folding were studied. Figure 3e shows the EPSC behavior stimulated by a light pulse in flat, bending, and folding states. The current at 0 and 10 s after light removal remains unchanged under different bending states. To further examine the stability and bending endurance, the variation of EPSC value from initial to 1000 bending cycles with an interval of 10 cycles was demonstrated, as shown in Figure 3f. The current at 0 and 10 s after light removal was kept very well, exhibiting stable and repeatable cycling performance without significant performance degradation. The results show the robust flexibility of the device, demonstrating the potential for mimicking human visual perception. On this basis, candy‐shaped lights with the same light intensity and different durations (2, 20, 40 s) are predefined into the array to realize the information storage. As shown in Figure 3b, the current increases with the duration time, indicating that a stronger memory effect can be obtained, which is consistent with the previous results.^[^ ^47^, ^48^ ^]^ It is worth noting that the candy‐shaped pattern can be clearly distinguished even under the short‐term light of 2 s. The upper six pictures of Figure S14 in the Supporting Information and Figure 3b show the current change with duration time when the decay time is 60 and 300 s, respectively. As the decay time increase, the current will be gradually decreased to a low level which will not be sufficient for image recognition. The results mean that the memory strength increases with the duration time and decreases with the decay time, which is consistent with the human visual perception.

**Figure 4:**
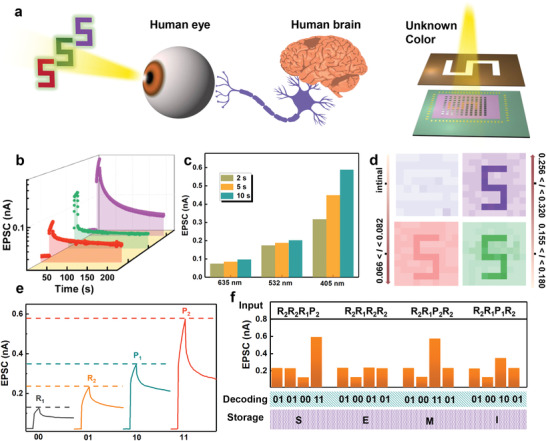
a) Schematic diagram of the human visual system and a 10 × 10 array device that recognizes unknown light. b) EPSC is triggered by light of different wavelength with the same intensity (0.1 mW cm^−2^, duration of 10 s). c) Current value of different wavelength at different duration time of 2, 5, and 10 s. d) The recognition results in the dark condition and illumination at 635, 532, and 405 nm for 2 s. Unit is nA. e) Define the light pulses of different wavelengths and intensities to decode the two‐digit information of 00 (635 nm, 0.1 mW cm^−2^; *R*_1_), 01 (635 nm, 0.5 mW cm^−2^; *R*_2_), 10 (405 nm, 0.1 mW cm^−2^; *P*_1_), and 11 (405 nm, 0.5 mW cm^−2^; *P*_2_). f) The decode procedure of the device according to the ASCII code.

**Figure** **4**a shows the schematic diagram of the human visual system and the device that recognizes unknown light. According to the absorption spectra, the absorption layer exhibits different sensitivity to light of the same power at 405, 532, and 635 nm, which is consistent with the different sensitivity of human eyes to different colors of visible light. Therefore, the potential of the artificial vision system to distinguish between purple, green, and red lights was studied. As shown in Figure 4b, 405, 532, and 635 nm light with the same power intensity is illuminated on the device (duration of 10 s), resulting in different EPSC levels. When the light wavelength are 405, 532, 635 nm, the observed current values are 588, 202, and 98 pA, respectively. Moreover, the current values at different wavelengths are distinguishable during the decay time, thus the device could distinguish the color even after the lights are turned off. This is the basis for the device to simulate the human eye to distinguish color.

Figure 4c summarizes the EPSC value for different duration times (2, 5, and 10 s). As expected, the current value increases with the duration time. The right side of Figure 4a shows the schematic diagram of the 10 × 10 array device that recognizes unknown light, when a light enters a mask that contains a letter “5,” a “5” pattern light is generated. The array device is illuminated by light of 635, 532, and 405 nm (duration of 2 s), respectively, with the same power intensity. And the photocurrent magnitudes measured by the devices are 66–82, 155–180, and 256–320 pA, respectively. The corresponding imaging results are shown in Figure 4d. The result reveals that the device can mimic the retinal function of the human eye by distinguishing colors. Furthermore, benefiting from the flexibility of the low‐dimensional properties of Cs_3_Bi_2_I_9_ NCs, the device exhibits stability during the bending and folding state. As shown in Figure S15 in the Supporting Information, after 1000 bending and folding cycles, the imaging results of letter “5” have almost no significant performance degradation, respectively (405 nm, 0.1 mW cm^−2^, duration of 2 s). In addition, by using the characteristic of both the wavelength‐ and intensity‐dependent response, an optical communication can be prepared. Here, the wavelength of the light is defined as the first digit information, and the light intensity is defined as the second digit information. The digit information can be transmitted and demodulated with light. Specifically, the wavelengths of 635 nm (red) and 405 nm (purple) represent 0 and 1 of the first digit information, and 0.1 and 0.5 mW cm^−2^ are demodulated to 0 and 1 of the second digit information, respectively. As shown in Figure 4e, the four pieces of two‐digit information of 00, 01, 10, and 11 can be demodulated and stored from the wavelength and intensity of the incident light, with the corresponding EPSC of 70 pA (red, 0.1 mW cm^−2^; *R*_1_), 130 pA (red, 0.5 mW cm^−2^; *R*_2_), 250 pA (purple, 0.1 mW cm^−2^; *P*_1_), 430 pA (purple, 0.5 mW cm^−2^; *P*_2_). As a result, according to the American Standard Code for Information Interchange (ASCII) rules, four light pulses *R*_2_ *R*_2_ *R*_1_ *P*_2_, *R*_2_ *R*_1_ *R*_2_ *R*_2_, *R*_2_ *R*_1_ *P*_2_ *R*_2_, and *R*_2_ *R*_1_ *P*_1_ *R*_2_, represent 01010011, 01000101, 01001101, and 01001001, respectively, can be demodulated into four letters “SEMI” and stored in the device. To avoid the interference between the adjacent light beams and to ensure the accurate demodulation of the optical signals, the device was erased with an electrical pulse after reading each light bit. It should be note that the functions of optical signal detection, decoding, simple calculation, and storage are realized, which can be used for multifunctional optoelectronic devices.

**Figure 5:**
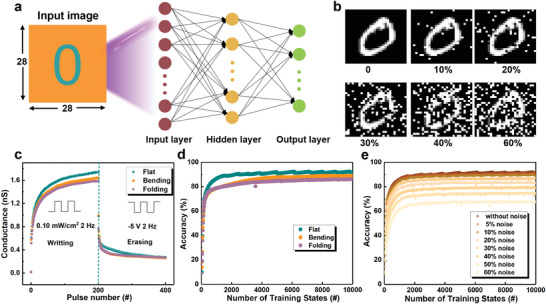
a) Schematic of an ANN for handwritten digits recognition with 28 × 28 pixels. b) Handwritten digits with different noise ratios. c) LTP/D curves of conductance state in flat, bending, and folding states. Recognition accuracy d) in different states and e) under different noise ratios in flat state.

To examine the potential of the device in the field of neuromorphic computing in the simulated human visual system, the long‐term potentiation (LTP) and long‐term depression (LTD) behavior of the device was analyzed to simulate the handwritten digit recognition task of the modified national institute of standards and technology (MNIST) database by an ANN. **Figure** **5**a shows the schematic diagram of a three‐layer perceptron ANN with one hidden layer, which depicts how the perceived image (28 × 28 pixels) is transmitted to the ANN to complete image training and recognition functions. The synaptic weight of the network is defined as the conductance difference between two equivalent optical synapses: *W* = *G* ^+^ − *G* ^−^.^[^ ^49^, ^50^, ^51^ ^]^ And the update of ANN parameters is implemented according to the synaptic plasticity (LTP and LTD), and the recognition accuracy is highly related to the linearity of the weight update trajectory. Figure 5c depicts the LTP and LTD curves in flat, bending, and folding states, in which 200 continuous optical spikes were applied to the device (405 nm, 2 Hz) and followed by another 200 negative voltage pulses (−5 V, 2 Hz). It can be observed that the conductance can be enhanced from 0.02 to 1.74 nS during the light‐writing process, and the conductance is reduced from 1.74 to 0.261 nS after voltage‐erasing process. The nonlinearities of the LTP and LTD curves are extracted from the following equations^[^ ^9^ ^]^

(1) $$G_{n+1}-G_{n}=\alpha_{P}e^{-\beta_{P}\frac{G_{n}-G_{\min}}{G_{\max}-G_{\min}}}\left(\mathrm{for}\;\mathrm{LTP}\right)$$

(2) $$G_{n+1}-G_{n}=-\alpha_{P}e^{-\beta_{P}\frac{G_{\max}-G_{n}}{G_{\max}-G_{\min}}}\left(\mathrm{for}\;\mathrm{LTD}\right)$$

where the *G_n_*_+1_ and *G_n_* represent the synaptic conductance of the device in the present and updated states, and the parameters *α* and *β* denotes the changing step sizes of the conductance and nonlinearity, respectively. *G*_max_ and *G*_min_ are the measured maximum and minimum values of *G*, respectively.^[^ ^9^, ^40^ ^]^ The fitted nonlinearities curve in flat state under 200 pulses is shown in Figure S16 in the Supporting Information, and parameters are calculated as 3.5174 and 3.5944, respectively, demonstrating an outstanding linearity for image recognition. In addition, the fitted parameters' result at bending states is listed in Table S1 in the Supporting Information. The result shows that the device still has good linearity even in bending state, which proves that the device possesses good stability. Based on the fitted parameters in the flat and bending states, the recognition accuracy is illustrated in Figure 5d. After the training process of 10 000 states, the recognition accuracy reached 0.921 (flat), 0.888 (bending), and 0.859 (folding), respectively. The small difference in recognition accuracy between flat and bending/folding states reveals that the device possesses great potential to fabricate hardware ANNs for flexible applications. Furthermore, different noise levels (0−60%) were implemented to the MNIST database to evaluate the fault tolerance of the ANNs (Figure 5b). As shown in Figure 5e and Figure S17 in the Supporting Information, the recognition accuracy is still higher than 80% when the noise ratio is set to 30%, demonstrating a strong fault‐tolerance ability.

## 3 Conclusions

In conclusion, the flexible artificial optoelectronic synaptic device based on Cs_3_Bi_2_I_9_ NCs/PMMA/DPPDTT FET has been demonstrated. Using the carrier confinement caused by the band mismatching between the Cs_3_Bi_2_I_9_ nanocrystals and the organic semiconductor layer, the device shows light‐induced charge trapping and electrical‐mediated charge release characteristics. The synaptic functions including EPSC, PPF, the transition from short‐term plasticity to long‐term plasticity, and “learning‐forgetting‐learning” were performed in our device. The artificial vision system is successfully simulated to achieve real‐time detection and image memory. In addition, benefiting from the low‐dimensional structure of the Cs_3_Bi_2_I_9_ NCs, the flexible array device demonstrates robust stability and reliability at bending states. Moreover, the neuromorphic computing of image recognition possess high accuracy and strong fault tolerance even in bending states. These results indicate that optoelectronic synaptic device with simple manufacturing technology of organic semiconductors and low‐dimensional Cs_3_Bi_2_I_9_ NCs are expected to play a vital role in the realization of neuromorphic computing in the future.

## 4 Experimental Section

### Materials

Cesium acetate (C_2_H_3_CsO_2_, 99.9%), bismuth acetate (C_6_H_9_BiO_6_, 99.9%), trimethylsilyl iodide (TMSI, 97%), and oleylamine (OAm, 80–90%) were provided from Aladdin. Oleic acid (OA, 90%) and 1‐octadecene (ODE, 90%) were provided by Sigma‐Aldrich. All the chemical reagents were commercially available and used as received.

### Preparation of Cs_3_Bi_2_I_9_ NCs

The Cs_3_Bi_2_I_9_ NCs were prepared by a modulated colloidal synthetic route. First, a mixture of 0.15 mmol C_2_H_3_CsO_2_, 0.1 mmol C_6_H_9_BiO_6_, 5.0 mL ODE, and 0.5 mL OA was placed in a three‐neck round‐bottom flask, and the solution was heated to 100 ℃ for 30 min. Then, the 90 µL TMSI was injected into the reaction mixture quickly and reaction terminated immediately after 10 s. Finally, the uniform hexagonal Cs_3_Bi_2_I_9_ nanocrystals were obtained.

### Fabrication of the PET/Al_2_O_3_/Cs_3_Bi_2_I_9_ NCs/PMMA/DPPDTT Device

First, the cleaned flexible PET substrate was treated with O_2_ gas plasma for 15 min. A laser writing method and thermal evaporation was used to fabricate the 10 × 10 array. The first electrode layer is patterned by laser writing method and is composed of 10 lines, the length is 2.5 cm, the width is 20 µm, and the spacing is 0.198 cm. Then, the Al_2_O_3_ film (20 nm) was deposited by atomic layer deposition at 200 °C. Second, the PMMA was dissolved in toluene with a concentration of 15 mg mL^−1^. The Cs_3_Bi_2_I_9_ NCs dispersed in toluene was mixed with PMMA solution and stirred for 24 h. The mixed solution was spin‐coated on the prepared substrate at 500 rpm for 5 s and then 3000 rpm for 30 s. Then, the samples were annealed at 60 ℃ for 30 min. Third, The DPPDTT was dissolved in dichlorobenzene with a concentration of 3 mg mL^−1^ and then was spin‐coated on the substrate at 3000 rpm for 30 s. Finally, the metal plate nested with the gate was used as a mask, and the source and drain were prepared by thermal evaporation. All the electrode layer employed the metal stack of Cr/Au (5/50 nm).

### Statistical Analysis

The diameters of NCs in a typical TEM image were measured by software of Nano‐Measurement. The current statistics of array device were carried out via the origin2017 software. The sample size for all current statistics was 100.

### Characterization of Materials and Devices

The crystal structure of the prepared Cs_3_Bi_2_I_9_ NCs was measured by HRTEM (JEM‐2010F). XRD (Rigaku D/Max‐2550) was performed to study the crystallinity of the sample. The PL spectra were studied by a spectrofluorometer (Horiba; Fluorolog‐3). The absorption spectra were conducted by a Shimadzu UV‐3150 spectrophotometer. The electrical characteristics were conducted using a Keithley 4200‐SCS parameter analyzer. The light source was power‐adjustable. UTG4082A were conducted to provide a pulse signal to generate pulsed light. All the device tests were carried out under an ambient condition at room temperature. The image processing was carried out using MATLAB.

### Neural Network Stimulation

The structure of ANN including input layer, hidden layer, and output layer was used. The number of neurons of three layers was 784, 400, 10, respectively. The algorithm utilized for training and recognition processes was back‐propagation (BP) algorithm. A total of 50 000 images were applied for training, and 10 000 images were used for testing the accuracy.

## Conflict of Interest

The authors declare no conflict of interest.

## Supporting information

Supporting Information

## Data Availability

The data that support the findings of this study are available from the corresponding author upon reasonable request.

## References

[1] B. J. Shastri , A. N. Tait , T. Ferreira de Lima , W. H. P. Pernice , H. Bhaskaran , C. D. Wright , P. R. Prucnal , Nat. Photonics 2021, 15, 102.

[2] C. Liu , X. Yan , X. Song , S. Ding , D. W. Zhang , P. Zhou , Nat. Nanotechnol. 2018, 13, 404. 29632398 10.1038/s41565-018-0102-6

[3] Y. X. Hou , Y. Li , Z. C. Zhang , J. Q. Li , D. H. Qi , X. D. Chen , J. J. Wang , B. W. Yao , M. X. Yu , T. B. Lu , J. Zhang , ACS Nano 2021, 15, 1497. 33372769 10.1021/acsnano.0c08921

[4] S. Manipatruni , D. E. Nikonov , I. A. Young , Nat. Phys. 2018, 14, 338.

[5] S. Seo , B. S. Kang , J. J. Lee , H. J. Ryu , S. Kim , H. Kim , S. Oh , J. Shim , K. Heo , S. Oh , J. H. Park , Nat. Commun. 2020, 11, 3936. 32769980 10.1038/s41467-020-17849-3 PMC7414205

[6] T. Y. Wang , J. L. Meng , Z. Y. He , L. Chen , H. Zhu , Q. Q. Sun , S. J. Ding , P. Zhou , D. W. Zhang , Adv. Sci. 2020, 7, 1903480. 10.1002/advs.201903480 PMC7175259 32328430

[7] K. Lu , X. Li , Q. Sun , X. Pang , J. Chen , T. Minari , X. Liu , Y. Song , Mater. Horiz. 2021, 8, 447. 34821264 10.1039/d0mh01520b

[8] M. Hu , C. E. Graves , C. Li , Y. Li , N. Ge , E. Montgomery , N. Davila , H. Jiang , R. S. Williams , J. J. Yang , Q. Xia , J. P. Strachan , Adv. Mater. 2018, 30, 1705914. 10.1002/adma.201705914 29318659

[9] J. Sun , S. Oh , Y. Choi , S. Seo , M. J. Oh , M. Lee , W. B. Lee , P. J. Yoo , J. H. Cho , J. H. Park , Adv. Funct. Mater. 2018, 28, 1804397.

[10] Y. Shen , N. C. Harris , S. Skirlo , M. Prabhu , T. Baehr‐Jones , M. Hochberg , X. Sun , S. Zhao , H. Larochelle , D. Englund , M. Soliacic , Nat. Photonics 2017, 11, 441.

[11] J. Kim , H. C. Lee , K. H. Kim , M. S. Hwang , J. S. Park , J. M. Lee , J. P. So , J. H. Choi , S. H. Kwon , C. J. Barrelet , H. G. Park , Nat. Nanotechnol. 2017, 12, 963. 28785091 10.1038/nnano.2017.153

[12] Y. C. Chiang , C. C. Hung , Y. C. Lin , Y. C. Chiu , T. Isono , T. Satoh , W. C. Chen , Adv. Mater. 2020, 32, 2002638. 10.1002/adma.202002638 32700349

[13] X. H. Wu , Y. L. Chu , R. Liu , H. E. Katz , J. Huang , Adv. Sci. 2017, 4, 1700442. 10.1002/advs.201700442 PMC5737237 29270350

[14] J. L. Shi , J. S. Jie , W. Deng , G. Luo , X. C. Fang , Y. L. Xiao , Y. J. Zhang , X. J. Zhang , X. H. Zhang , Adv. Mater. 2022, 34, 2200380. 10.1002/adma.202200380 35243701

[15] H. W. Tan , G. Liu , X. J. Zhu , H. L. Yang , B. Chen , X. X. Chen , J. Shang , W. D. Lu , Y. H. Wu , R. W. Li , Adv. Mater. 2015, 27, 2797. 25786781 10.1002/adma.201500039

[16] H. W. Tan , G. Liu , H. L. Yang , X. H. Yi , L. Pan , J. Shang , S. N. Long , M. Liu , Y. H. Wu , R. W. Li , ACS Nano 2017, 11, 11298. 29028312 10.1021/acsnano.7b05762

[17] F. C. Zhou , Z. Zhou , J. W. Chen , T. H. Choy , J. L. Wang , N. Zhang , Z. Y. Lin , S. M. Yu , J. F. Kang , H. S. P. Wong , Y. Chai , Nat. Nanotechnol. 2019, 14, 776. 31308498 10.1038/s41565-019-0501-3

[18] M. Lee , W. Lee , S. Choi , J. Jo , J. Kim , S. K. Park , Y. Kim , Adv. Mater. 2017, 29, 1700951. 10.1002/adma.201700951 28514064

[19] S. C. Qin , F. Q. Wang , Y. J. Liu , Q. Wan , X. R. Wang , Y. B. Xu , Y. Shi , X. M. Wang , R. Zhang , 2D Mater. 2017, 4, 035022.

[20] Z. C. Zhang , Y. Li , J. J. Wang , D. H. Qi , B. W. Yao , M. X. Yu , X. D. Chen , T. B. Lu , Nano Res. 2021, 14, 4591.

[21] M. J. Xu , T. F. Xu , A. Q. Yu , H. L. Wang , H. Wang , M. Zubair , M. Luo , C. Shan , X. G. Guo , F. Wang , W. D. Hu , Y. M. Zhu , Adv. Opt. Mater. 2021, 9, 2100937.

[22] S. Seo , S. H. Jo , S. Kim , J. Shim , S. Oh , J. H. Kim , K. Heo , J. W. Choi , C. Choi , S. Oh , D. Kuzum , H. S. P. Wong , J. H. Park , Nat. Commun. 2018, 9, 5106. 30504804 10.1038/s41467-018-07572-5 PMC6269540

[23] Z. H. Dai , S. K. Yadavalli , M. Chen , A. Abbaspourtamijani , Y. Qi , N. P. Padture , Science 2021, 372, 618. 33958474 10.1126/science.abf5602

[24] W. Q. Wu , X. D. Wang , X. Han , Z. Yang , G. Y. Gao , Y. F. Zhang , J. F. Hu , Y. W. Tan , A. L. Pan , C. F. Pan , Adv. Mater. 2018, 31, 1805913.

[25] Y. Wang , Z. Y. Lv , J. R. Chen , Z. P. Wang , Y. Zhou , L. Zhou , X. L. Chen , S. T. Han , Adv. Mater. 2018, 30, 1802883. 10.1002/adma.201802883 30063261

[26] J. T. Chen , Y. C. Chui , Y. T. Li , C. C. Chueh , W. C. Chen , Adv. Mater. 2017, 29, 1702217.

[27] Y. H. Chang , C. W. Ku , Y. H. Zhang , H. C. Wang , J. Y. Chen , Adv. Funct. Mater. 2020, 30, 2000764.

[28] W. K. Lin , G. X. Chen , E. L. Li , L. H. He , W. J. Yu , G. Peng , H. P. Chen , T. L. Guo , ACS Appl. Mater. Interfaces 2020, 12, 43967. 32867472 10.1021/acsami.0c12185

[29] Y. L. Sun , L. Qian , D. Xie , Y. X. Lin , M. X. Sun , W. W. Li , L. M. Ding , T. L. Ren , T. Palacios , Adv. Funct. Mater. 2019, 29, 1902538.

[30] L. Qian , Y. L. Sun , M. M. Wu , C. Li , D. Xie , L. M. Ding , G. Q. Shi , Nanoscale 2018, 10, 6837. 29616272 10.1039/c8nr00914g

[31] Y. C. Cheng , H. Li , B. Liu , L. Jiang , M. Liu , H. Huang , J. L. Yang , J. He , J. Jiang , Small 2020, 16, 2005217. 10.1002/smll.202005217 33035390

[32] Q. B. Zhu , B. Li , D. D. Yang , C. Liu , S. Feng , M. L. Chen , Y. Sun , Y. N. Tian , X. Su , X. M. Wang , S. Qiu , Q. W. Li , X. M. Li , H. B. Zeng , H. M. Cheng , D. M. Sun , Nat. Commun. 2021, 12, 1798. 33741964 10.1038/s41467-021-22047-w PMC7979753

[33] D. D. Hao , J. Y. Zhang , S. L. Dai , J. H. Zhang , J. Huang , ACS Appl. Mater. Interfaces 2020, 12, 39487. 32805934 10.1021/acsami.0c10851

[34] H. Tian , L. Zhao , X. Wang , Y. W. Yeh , N. Yao , B. P. Rand , T. L. Ren , ACS Nano 2017, 11, 12247. 29200259 10.1021/acsnano.7b05726

[35] X. J. She , D. Gustafsson , H. Sirringhaus , Adv. Mater. 2017, 29, 1604769. 10.1002/adma.201604769 28004860

[36] G. M. Paternò , N. Mishra , A. J. Barker , Z. Dang , G. Lanzani , L. Manna , A. Petrozza , Adv. Funct. Mater. 2019, 29, 1805299.

[37] Z. Z. Ma , Z. F. Shi , L. T. Wang , F. Zhang , D. Wu , D. W. Yang , X. Chen , Y. Zhang , C. X. Shan , X. J. Li , Nanoscale 2020, 12, 3637. 32016263 10.1039/c9nr10075j

[38] W. G. Li , X. D. Wang , J. F. Liao , Y. Jiang , D. B. Kuang , Adv. Funct. Mater. 2020, 30, 1909701.

[39] Z. Y. Qi , X. W. Fu , T. F. Yang , D. Li , P. Fan , H. L. Li , F. Jiang , L. H. Li , Z. Y. Luo , X. J. Zhuang , A. L. Pan , Nano Res. 2019, 12, 1894.

[40] X. Hou , H. Zhang , C. Liu , S. Ding , W. Bao , D. W. Zhang , P. Zhou , Small 2018, 14, 1800319. 10.1002/smll.201800319 29665261

[41] Y. X. Zhang , Y. C. Liu , Z. Xu , H. C. Ye , Z. Yang , J. X. You , M. Liu , Y. H. He , M. G. Kanatzidis , S. Z. Liu , Nat. Commun. 2020, 11, 2304. 32385231 10.1038/s41467-020-16034-w PMC7210296

[42] F. Gao , M. L. Song , M.‐C. Wong , R. Ding , W. F. Lo , S.‐Y. Pang , W. J. Jie , J. H. Hao , Adv. Funct. Mater. 2021, 32, 2108014.

[43] D. Choquet , A. Triller , Neuron 2013, 80, 691. 24183020 10.1016/j.neuron.2013.10.013

[44] T. S. Zhao , C. Zhao , W. Y. Xu , Y. Liu , H. Gao , I. Z. Mitrovic , E. G. Lim , L. Yang , C. Z. Zhao , Adv. Funct. Mater. 2021, 31, 2106000.

[45] L. D. Li , S. Ye , J. L. Qu , F. F. Zhou , J. Song , G. Z. Shen , Small 2021, 17, 2005606. 10.1002/smll.202005606 33728799

[46] Y. Chen , X. Yang , P. Sun , W. Dou , X. Chen , C. Zhang , C. Shan , Mater. Horiz. 2021, 8, 3368. 34664595 10.1039/d1mh01304a

[47] W. X. Wang , S. Gao , Y. Li , W. J. Yue , H. Kan , C. W. Zhang , Z. Lou , L. L. Wang , G. Z. Shen , Adv. Funct. Mater. 2021, 31, 2101201.

[48] J. Y. Du , D. G. Xie , Q. H. Zhang , H. Zhong , F. Q. Meng , X. K. Fu , Q. C. Sun , H. Ni , T. Li , E. Guo , H. Z. Guo , M. He , C. Wang , L. Gu , X. L. Xu , G. Y. Zhang , G. Z. Yang , K. J. Jin , C. Ge , Nano Energy 2021, 89, 106439.

[49] X. F. Lu , Y. S. Zhang , N. Z. Wang , S. Luo , K. L. Peng , L. Wang , H. Chen , W. B. Gao , X. H. Chen , Y. Bao , G. Liang , K. P. Loh , Nano Lett. 2021, 21, 8800. 34644096 10.1021/acs.nanolett.1c03169

[50] J. X. Wang , Y. Chen , L. A. Kong , Y. Fu , Y. L. Gao , J. Sun , Appl. Phys. Lett. 2018, 113, 151101.

[51] J. Rao , Z. Fan , L. Hong , S. Cheng , Q. Huang , J. Zhao , X. Xiang , E. J. Guo , H. Guo , Z. Hou , Y. Chen , X. Lu , G. Zhou , X. Gao , J. M. Liu , Mater. Today Phys. 2021, 18, 100392.

