# Supplementary material for: Flexible Artificial Optoelectronic Synapse based on Lead‐Free Metal Halide Nanocrystals for Neuromorphic Computing and Color Recognition
Source: Adv Sci (Weinh). 2022 Jun 5;9(22):2202123. doi: 10.1002/advs.202202123 (PMC9353487; doi:10.1002/advs.202202123)
Supplement: Supplementary file 1 — Supporting Information [file ADVS-9-2202123-s001.pdf]

ADVANCED SCIENCE
Open Access

Supporting Information

for *Adv. Sci.*, DOI: 10.1002/advs.202202123

Flexible Artificial Optoelectronic Synapse based on Lead-Free Metal Halide Nanocrystals for Neuromorphic Computing and Color Recognition

*Ying Li, Jiahui Wang, Qing Yang, and Guozhen Shen**

Copyright WILEY-VCH Verlag GmbH & Co. KGaA, 69469 Weinheim, Germany, 2022.

Supporting Information

Flexible Artificial Optoelectronic Synapse Based on Lead-Free Metal Halide Nanocrystals for Neuromorphic Computing and Color Recognition

*Ying Li, Jiahui Wang, Qing Yang, Guozhen Shen**

Dr. Ying Li, Prof. Guozhen Shen
State Key Laboratory for Superlattices and Microstructures Institute of Semiconductors
Beijing 100083, China

Prof. Guozhen Shen
School of Integrated Circuits and Electronics, Beijing Institute of Technology, Beijing 100081, China.
E-mail: gzshen@bit.edu.cn

Dr. Jiahui Wang, Prof. Qing Yang
Laboratory of Nanomaterials for Energy Conversion, University of Science and Technology of China, Hefei 230026, P. R. China

Keywords: lead-free, $Cs_3Bi_2I_9$, flexible artificial synapse, neuromorphic computing, color recognition

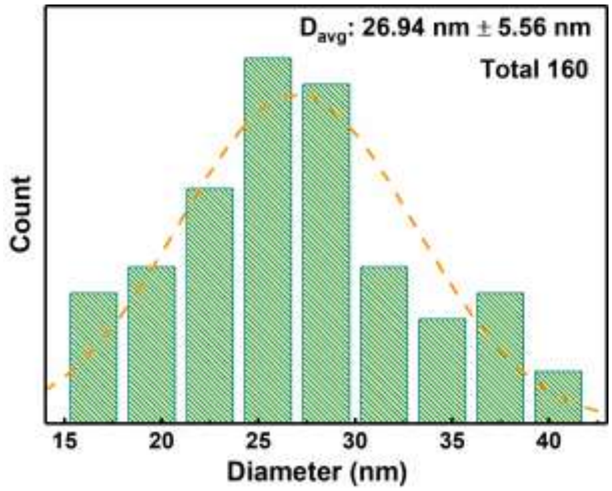

Figure S1. Statistical results of the diameter of $Cs_3Bi_2I_9$ NCs.

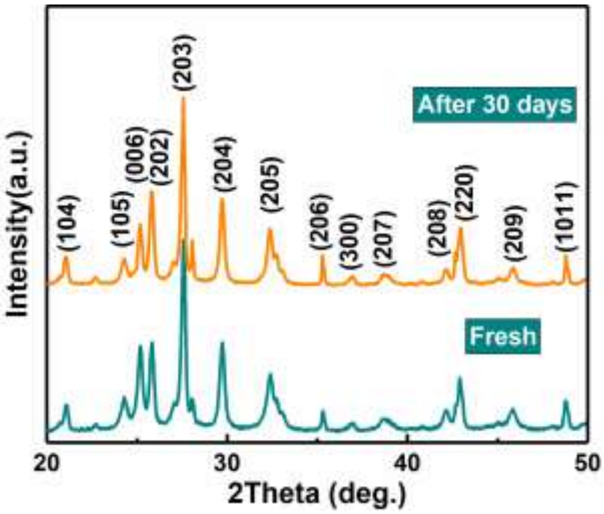

Figure S2. Comparison of the XRD patterns of the $Cs_3Bi_2I_9$ nanocrystals before and after storage for 30 days in air ambient.

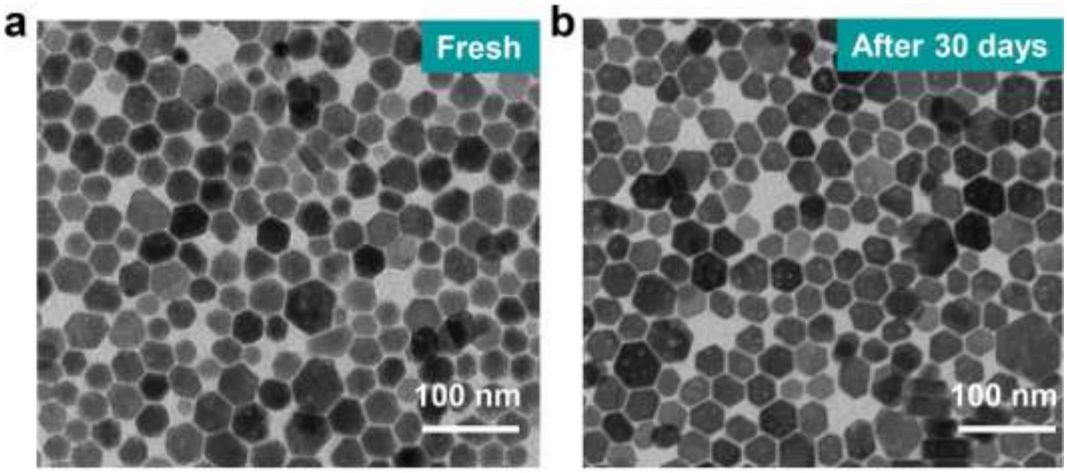

Figure S3. TEM images of a) fresh and b) stored in ambient conditions after 30 days.

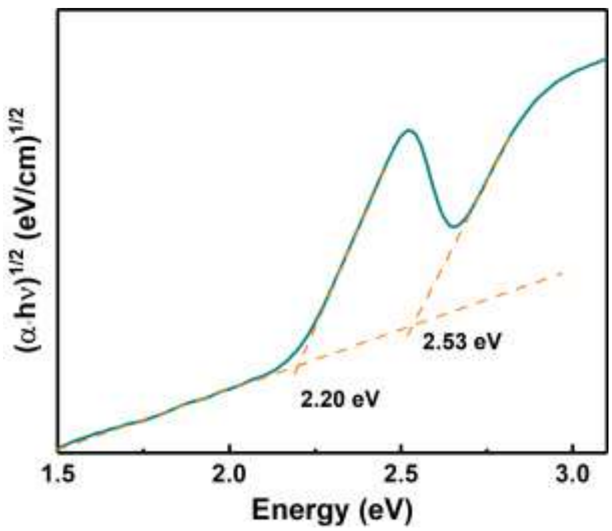

Figure S4 Calculation of the optical bandgap of $Cs_3Bi_2I_9$ nanocrystals using the Tauc method.

The bandgap of $Cs_3Bi_2I_9$ nanocrystals (indirect bandgap) was estimated using Tauc equation $(\alpha\cdot h\nu)^{1/2} = A(h\nu - E_g)$. And the value of indirect optical and electronic bandgaps for the as-grown $Cs_3Bi_2I_9$ nanocrystals is estimated to be around 2.20 eV and 2.53 eV.

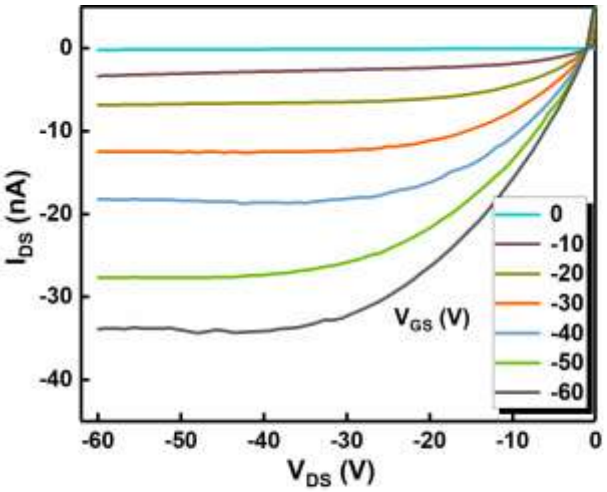

Figure S5. Output characteristics of the device in the dark condition with different V_{GS} values.

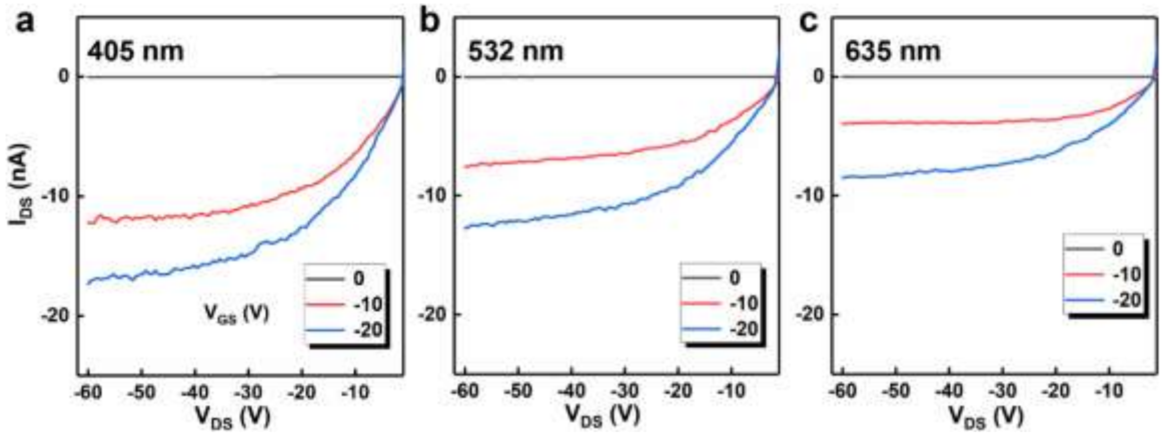

Figure S6. Output characteristics of $Cs_3Bi_2I_9$ QDs/PMMA/DPPDTT FETs under a) 405 nm, b) 532 nm, c) 635 nm light condition (0.1 mW cm^{-2}).

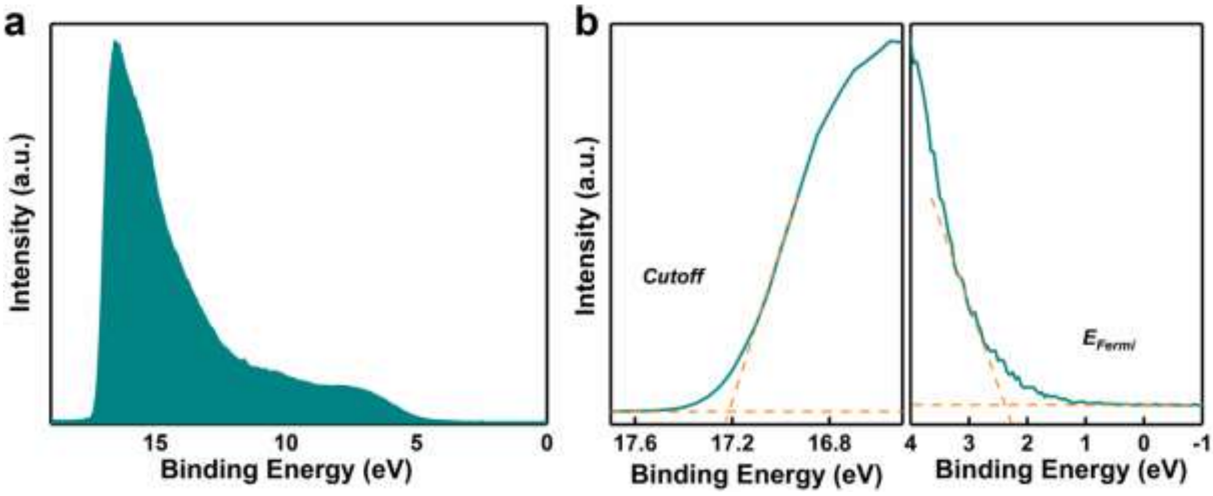

Figure S7 UPS data of the $Cs_3Bi_2I_9$ nanocrystals.

UPS measurements were performed to determine the valence band maximum of $Cs_3Bi_2I_9$ nanocrystals, and the value can be obtained by the following formulas

$$E_{VBM}=hv-E_{cutoff}+E_{Fermi} \quad (1)$$

$$E_{CBM}=E_{VBM}+E_g \quad (2)$$

in which hv is the ultraviolet radiation energy (21.22 eV), E_{cutoff} is the binding energy of the secondary cutoffs in the spectra, and E_{Fermi} is the difference between the valence band maximum (E_{VBM}) and the Fermi level. Combined with the bandgap energy of 2.20 eV, we derived the E_{VBM} and conduction band minimum (E_{CBM}) value as 6.39 eV and 4.19 eV, respectively.

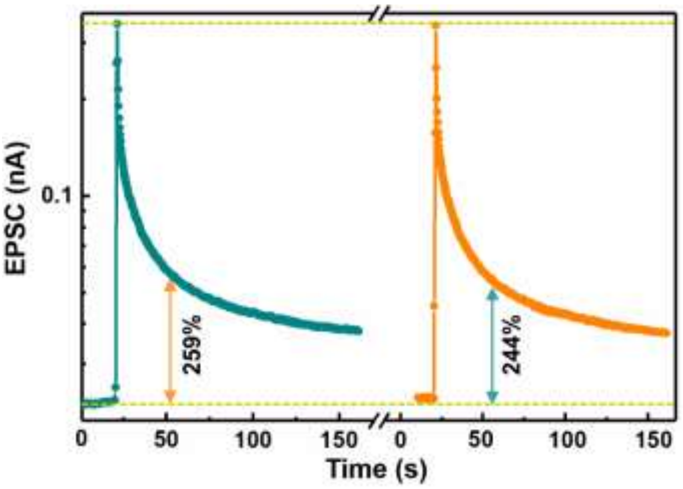

Figure S8. Stability of the unencapsulated device after 1 month storage in the open air (405 nm, 0.1 mW cm^{-2}, duration of 1 s).

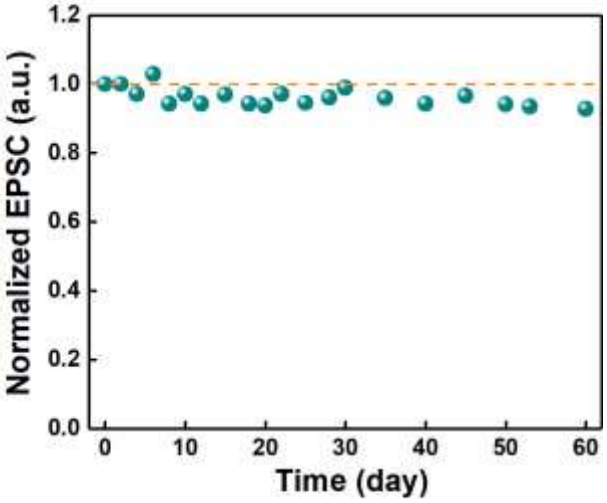

Figure S9. Stability of the unencapsulated device after 60 days storage in the open air (405 nm, 0.1 mW cm^{-2}, duration of 1 s).

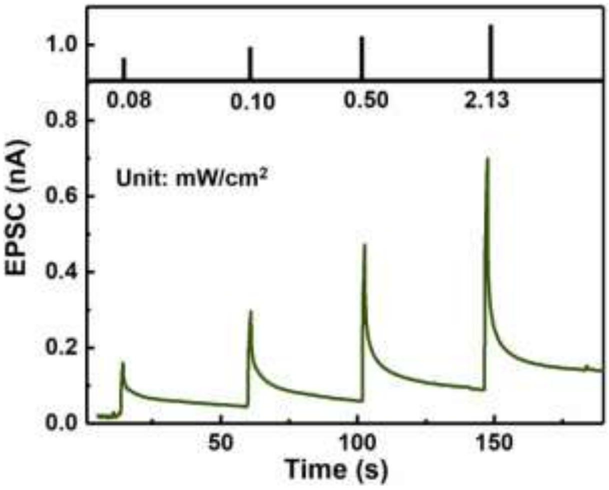

Figure S10. EPSC behavior triggered by light pulses of different intensities (pulse width is 1 s).

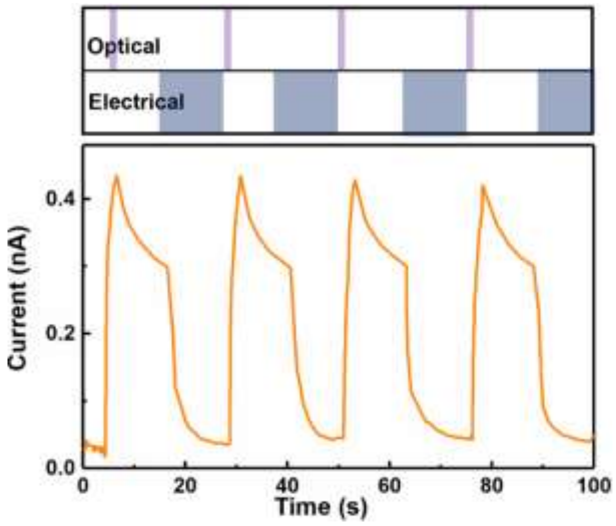

Figure S11. Switching characteristics of the device under a 405 nm light (0.10 mW/cm^2, pulse width 3 s) and a reset voltage pulse (V_{GS}=−20 V, pulse width 10 s).

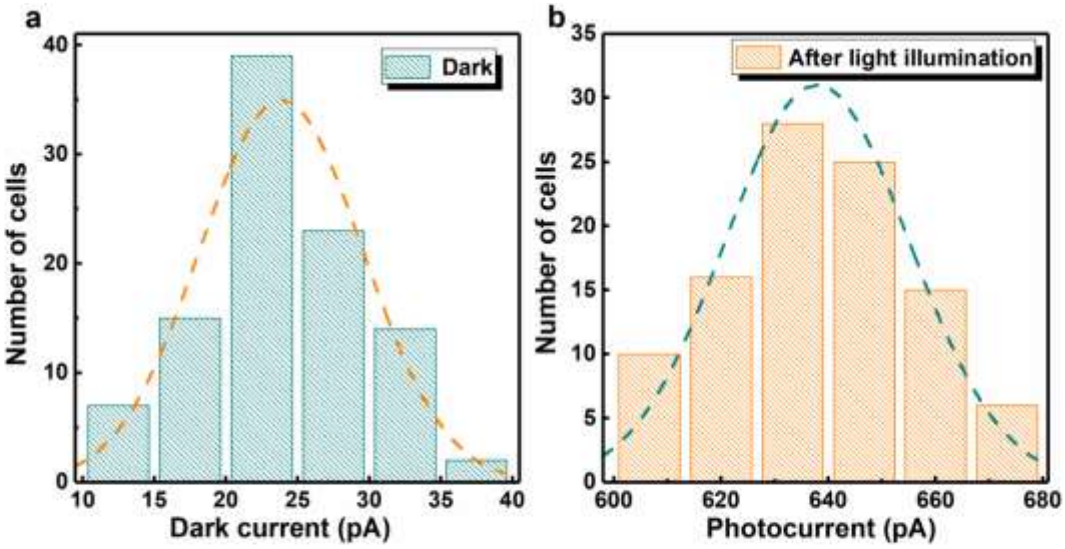

Figure S12. The statistical dark current and photocurrent after light illumination of 40 s (V_{GS}=0 V, V_{DS}=−10 V, 0.1 mW cm^{-2}). The sample size is 100.

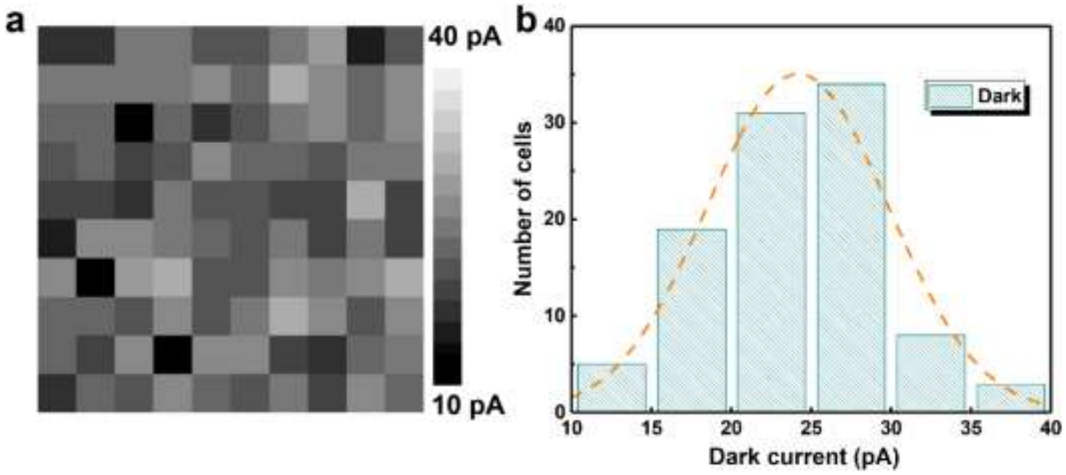

Figure S13. a) The dark current mapping of the device after electrical erasing (V_{GS}=−20 V, pulse width 10 s). b) The corresponding statistical dark current, and the sample size is 100.

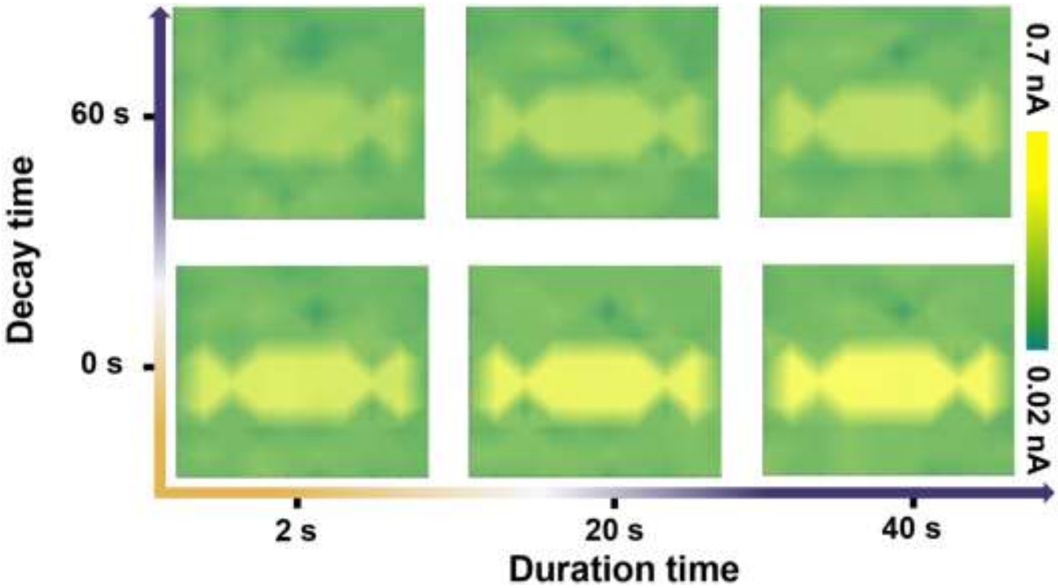

Figure S14. The current response image mapping of the 10 × 10 array with decay time of 0 s and 60 s.

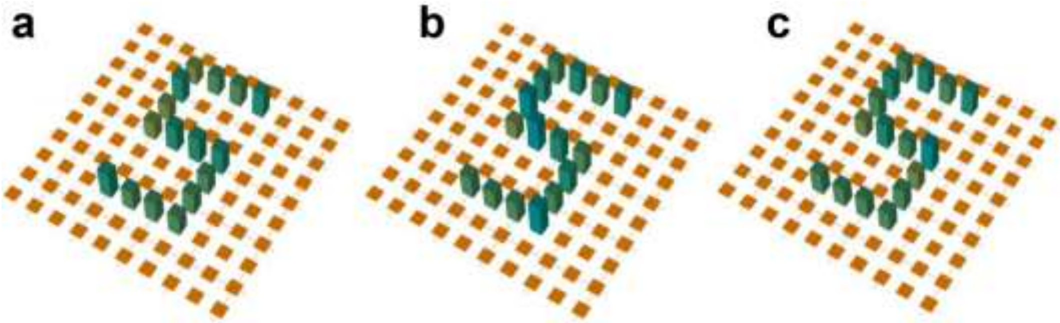

Figure S15. The imaging results of letter “5” in a) flat state and after 1000 b) bending and c) folding cycles (405 nm, 0.1 mW/cm^2, duration of 2 s).

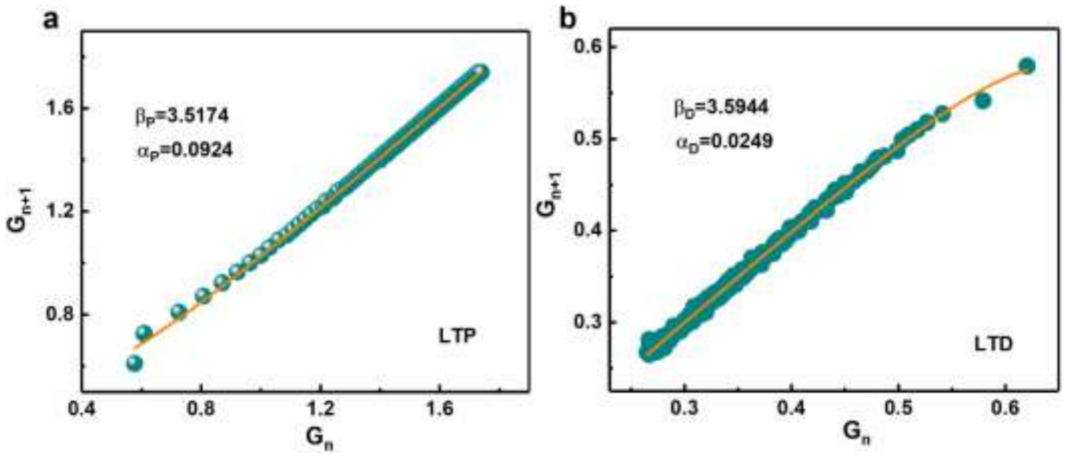

Figure S16. Fitting curves obtained from the experimental a) LTP data, and b) LTD data.

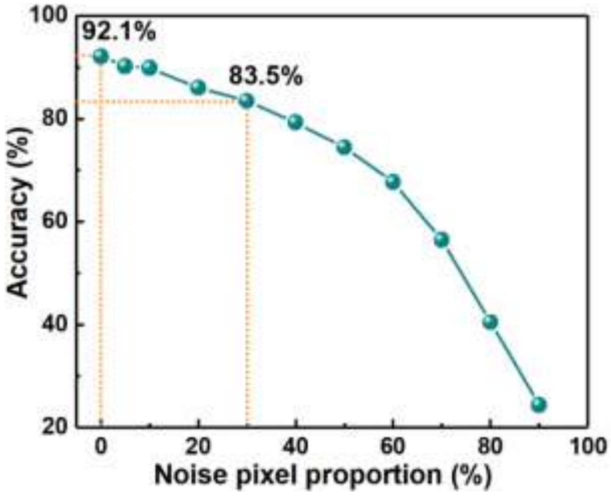

Figure S17. Recognition accuracy of the network as a function of noise pixel proportion.

Table S1 Extracted fitting parameters for the device at flat, bending, and folding state

State	α_P	β_P	α_D	β_D
**Flat**	0.0924	3.5174	0.0249	3.5944
**Bending**	0.0733	3.6178	0.0457	3.3416
**Folding**	0.0747	3.6517	0.0301	3.6241
